# Trends in Bullying and Emotional and Behavioral Difficulties Among Pakistani Schoolchildren: A Cross-Sectional Survey of Seven Cities

**DOI:** 10.3389/fpsyt.2019.00976

**Published:** 2020-01-17

**Authors:** Sadiq Naveed, Ahmed Waqas, Zarnain Shah, Waqas Ahmad, Muhammad Wasim, Jawaria Rasheed, Tayyaba Afzaal

**Affiliations:** ^1^ Department of Psychiatry and Behavioral Sciences, University of Kansas Medical Center, Kansas City, KS, United States; ^2^ Department of Psychiatry, CMH Lahore Medical College & Institute of Dentistry, Lahore Cantt, Pakistan; ^3^ Maternal and Child Mental Health, Human Development Research Foundation, Rawalpindi, Pakistan; ^4^ Department of Critical Care, Aga Khan University, Karachi, Pakistan; ^5^ Department of internal Medicine, Punjab Medical University, Faisalabad, Pakistan; ^6^ Department of General Surgery, Nishtar Medical University, Multan, Pakistan; ^7^ Department of Obstetrics and Gynecology, Fatima Jinnah Women Hospital, Multan, Pakistan; ^8^ Department of Psychology, Government College University, Lahore, Pakistan

**Keywords:** bullying, perpetration, victimization, Pakistan, children, behavioral problems, emotional problems

## Abstract

**Background:** Bullying and peer victimization among adolescents are growing public health concerns that affect victims’ emotional well-being, and their social and academic functioning. Despite concerns about this public health epidemic in low- and middle-income countries, most prevalence, policy and intervention studies have been conducted in developed countries and economies.

**Methods:** This cross-sectional study was conducted between September 2016 and July 2017 at seven public and private schools located in five districts in Pakistan: Lodhran, Rahim Yar Khan, Bahawalpur, Faisalabad, Multan, Thatta, and Nawabshah. A total of 2,315 schoolchildren were surveyed with a specially designed instrument in Urdu with items about demographics and bullying behavior, together with a strengths and difficulties questionnaire.

**Results:** Mean age of the respondents was 14.63 (2.87) years. More than half of the respondents were males (n = 1301, 56.2%), and a majority reported that their mothers were housewives (n = 2,100, 90.7%). A total of 615 (26.6%) respondents reported being bullied at school, and 415 (17.9%) reported being bullied away from school. Perpetration of bullying was reported by 430 (18.6%) participants at school and 376 (16.3%) away from school. Being bullied in the past was strongly associated with becoming a perpetrator of bullying in the future. Internalizing symptoms were significantly associated with male gender, older age, being a victim of bullying, and employment status of the respondent’s mother. Externalizing symptoms were significantly associated with male gender, older age, being a victim and perpetrator of bullying, and mother’s employment status.

**Conclusion:** Bullying perpetrators have a greater tendency to exhibit externalizing symptoms, whereas victims develop both externalizing and internalizing psychopathologies.

## Introduction

Bullying and peer victimization among adolescents are growing public health concerns because they affect victims’ emotional well-being, as well as their social and academic functioning (1). Although some of its characteristics overlap with other forms of aggression such as harassment, bullying is defined as unwanted and repetitive aggressive behaviors committed by a young individual or a group of young people towards the victim, and a perceived sense of power imbalance in favor of the aggressors (2). Unfortunately, bullying has traditionally been socially accepted as a “rite of passage” and a “developmentally appropriate” behavior among children and adolescents, despite its adverse psychological and social consequences (3). This unhealthy acceptance of bullying is also prevalent in the media and literature (4, 5).

Bullying usually occurs in settings such as classrooms or among siblings at home (2, 3, 6). Siblings or dating partners, however, are not seen as pivotal pillars in most operational definitions of bullying behaviors (2). Bullies try to assert their power over individuals with an emotional response to bullying or with limited social support (6). This epidemic is prevalent across all societies, ethnicities, races, cultures and geographies (3). The world-wide prevalence of bullying is estimated to be slightly more than 1 in 3 students aged 13 to 15 years (7), and these figures are substantiated by a recent metaanalysis of 80 studies that reported the global prevalence rate of traditional bullying to be 35% (8). Similar prevalence figures have been reported from developing countries such as Pakistan, where a nationally representative school-based health survey in 2009 estimated the prevalence of bullying behavior to be around 41% (9). These rates are significantly higher than those reported in the developed world, and the difference is substantiated by the 2011 CDC Youth Risk Behavior Survey, which estimated the prevalence of bullying among adolescents in the USA to be around 27% (10).

Bullying has also been associated with adverse physical, mental and academic consequences in children and adolescents. For example, victims of bullying were more likely to have a higher risk of depression, suicidal ideation, anxiety, sleep disturbances, social isolation and feelings of loneliness (11–14). Moreover, the prevalence of substance use including alcohol, nicotine and inhalants was reportedly higher among victims of bullying than non-victims (12, 15, 16). The perpetration of bullying is also associated with externalizing symptoms such as aggression, truancy and delinquency (11–14). A recent metaanalysis of 14 studies suggested a dose-dependent relationship between bullying and the development of psychotic symptoms—a finding that highlights the debilitating consequences of bullying (17, 18).

Somatic symptomatology and academic underachievement have also been associated with bullying behavior among children (3, 19, 20). A recent study of a sample Pakistani children reported that more than 89% of the young respondents reported one or more occurrences of victimization, and more than 75% reported one or more acts of perpetration (21). A high prevalence of bullying behavior among Pakistani youth was found to correlate significantly with hunger, absences from school, witnessing fights between the father and another man, and patriarchal attitudes (22). Further to these findings, a deeper investigation into gender roles and violence against women and girls among youth also demonstrated that the aforementioned factors along with bullying behavior also account for acceptance of patriarchal attitudes in Pakistan (23). However, only a few high-quality studies exploring the public health aspects of bullying have been conducted in this country. Most of these studies explored bullying in the context of patriarchal and gender-biased aspects of Pakistani society (22, 23), and this research reported participants’ gender to be a significant predictor of bullying behaviors. Compared to females, males were reported to be more involved in the perpetration of bullying, aggression and violence (22, 23). However, none of the studies compared the employment status of mothers or her availability at home as a possible correlate of bullying and emotional problems in children.

Recent years have seen improvements in our understanding of the psychosocial correlates of bullying behavior, owing to the progress made in data sciences. The psychosocial model of bullying behavior is viewed as complex due to potential overlaps in the frequency of involvement, perpetration and victimization among youth (24, 25). This approach reflects the view that psychosocial and health-related behaviors tend to cluster together, rather than acting as single factors in determining behavior (26, 27). Moreover, these complexities indicate that variable-centered approaches may be inferior to person-centered analytic approaches such as cluster analysis. In contrast to variable-centered analysis, cluster analysis uses patterns of behaviors, experiences, demographics and health to identify different subgroups, their frequencies, and heterogeneity in associations (28). This approach thus provides a better method to elucidate actual behavioral patterns among respondents, and thus sheds light on the conceptual overlaps in bullying behavior (24, 25).

There is a paucity of data exploring the relationships between bullying and its emotional and behavioral consequences in Pakistan. Moreover, no research has explored this phenomenon with person-centered analytical approaches. Therefore, the present study was designed with the following aims:

- To explore the prevalence of bullying, victimization and bully-victimization behaviors among Pakistani schoolchildren.
- To explore the association of mothers’ employment status with bullying behaviors among children.
- To elucidate the strength of association of different bullying behaviors with strengths and difficulties among schoolchildren.
- To identify the mediating effects of pure victimization behavior on the association between externalizing symptomatology and pure perpetration.
- To identify the mediating effects of pure perpetration behavior on the association between internalizing symptomatology and pure victimization.
- To use an unsupervised machine learning technique in orde to identify clusters of emotional and behavioral difficulties associated with patterns of bullying behavior among schoolchildren.

## Methods

### Study Design

This cross-sectional study was conducted between September 2016 and July 2017 at seven public and private schools located in five districts in Pakistan: Lodhran, Rahim Yar Khan, Bahawalpur, Faisalabad, Multan, Thatta, and Nawabshah (29). The districts of Lodhran, Rahim Yar Khan, Faisalabad, Bahawalpur, and Multan are located in Punjab province, where the total population of 12,388,824 represents three major ethnicities, i.e. the Saraiki, Punjabi, and Urdu-speaking populations (29). The districts of Thatta and Nawabshah are located near the Indus River in Sindh province, where a total population of 13,55,131 represents a Sindhi and Urdu-speaking majority (29). Our study sample was representative of both urban and rural populations. However, all participating schools were selected based on convenience sampling methods.

### Sample Size

The total sample size was calculated with two methods. A minimum sample size of 664 was calculated with the following parameters: 5% margin of error, 99% confidence level, hypothetical population size of N = 20,000, and 50% response distribution. Previous studies have reported low to moderate effect sizes for associations between bullying behavior and emotional and behavioral difficulties in children (19, 30). Therefore, for low to moderate effect sizes, an alpha error probability of 5%, a statistical power of 99% and 5 predictors, a minimum sample size of 1,342 was found to be suitable for this study (GPower, v. 3.1).

### Operational Definitions

This study focused on three different patterns of bullying behaviors: pure perpetrators, pure victims, and bully-victims. Pure perpetrators were defined as those having a history of bullying other children. Pure victims were those who had a history of being bullied by other children. Children who reported a history of both perpetration and victimization were classified as bully-victims.

### Survey Procedure and Questionnaire

A team of local researchers was recruited to approach the administrative staff of the schools to seek their permission to conduct the study at their sites. Ethical approval for this study was granted by the Peoples University of Medical and Health Sciences for Women, Nawabshah, and was found to meet the ethical criteria of the Declaration of Helsinki (1964) and comparable standards. An informed consent form and detailed brochure outlining the objectives of the study were mailed to each participant’s parents. They were assured that all data would be anonymous and that no individual findings would be reported.

Thereafter, children enrolled at these schools and aged 10 to 17 years were approached through a convenience sampling method by a team of interviewers including their teachers and local researchers. Only those participants were recruited whose parents provided their written consent for their child to participate in the study. The study sample was not stratified based on the number of schools or potential study participants. A universal sampling technique was preferred, in which we approached all students enrolled at each school during the study period.

No incentives were provided to the parents or children taking part in the study. The children completed a three-part survey containing items on a) demographics, b) experiences of bullying and victimization, c) help-seeking behavior, and d) a strengths and difficulties questionnaire. All surveys were self-administered in the Urdu language, the national language of Pakistan. Members of the research team distributed the survey to students in their regular classrooms in the presence of their teachers, and responded to any questions or comments from the students. They were given ample time during regular classroom hours to complete the survey. These measures ensured a high response rate and high accuracy of the responses.

### Measures

The first section of the survey inquired about respondents’ demographic characteristics including age, gender, mother’s employment status, and background. The second section comprised 11 items regarding experiences of bullying and victimization as perceived by the participants. They were asked about the frequency of both victimization and perpetration of bullying behaviors. Four items sought details on bullying behavior at school and outside school, e.g. at private tuition centers, in the previous six months. The responses to these four items were recorded on a Likert-type scale ranging from “not at all” to “most days”. These items yielded good internal consistency, with a Cronbach’s alpha value of 0.820, and showed a unidimensional factor pattern that explained 66.1% of the variance according to exploratory factor analysis. Internal consistency for both the victimization (alpha = 0.74) and perpetration subscale (alpha = 0.77) was acceptable. Participants who had a positive history of both bullying victimization and perpetration were classified as bully-victims.

The next three items specified the gender of bullying perpetrators as boy, girl or group. These items were rated on a four-point Likert-like scale ranging from “not at all” to “most days”. The third section consisted of four items pertaining to help-seeking behavior. Out of these four items, two dichotomous questions asked whether the respondent had talked to or sought help from anyone regarding these experiences. The next question inquired about the relationship of the help provider to the respondent, i.e., teacher, parent, friend, sibling, or other. The last question asked respondents whether they sought help from parents, teachers, relatives, siblings, friends, or others.

The fourth section comprised the Strengths and Difficulties Questionnaire (SDQ), which has undergone extensive validation in samples of Pakistani children and adolescents. This instrument yields scores across several domains of emotional difficulties and strengths including emotional symptoms, conduct problems, hyperactivity, peer relationship problems, prosocial, externalizing, and internalizing symptoms (31). The scores were grouped into three categories based on established cut-off values for normal, borderline and abnormal. The ranges of possible scores for each section were: a) total difficulties (20–40), emotional difficulties (7–10), conduct problems (5–10), hyperactivity (7–10), peer problems (6–10), prosocial behavior (0–4), and impact score (2–10). The present study used the impact supplement of the SDQ to assess the frequency of distress among participants at home, in the context of their friendships, and during leisure and classroom activities (31).

### Statistical Analyses

All data were analyzed with SPSS v. 20 (IBM, Chicago, IL, USA). Categorical variables are reported here as frequencies, and quantitative variables are reported as the mean and standard deviation. A series of t-tests for independent samples quantified the association of gender and mother’s employment status with scores on the SDQ. Pearson’s correlation coefficient was calculated to analyze associations between age and SDQ subscales. Subsequently, a series of linear regressions were used to evaluate the associations between demographic characteristics and bullying behaviors with total SDQ scores, impact supplement scores, and internalizing and externalizing subscale scores. Before regression analysis, multicollinearity among variables was checked with the variance inflation factor (VIF) and tolerance (TOL) values. A value ≥10 for VIF and ≤0.2 for TOL for any predictor in the regression analysis was considered significant multicollinearity. Hierarchal regression was also used to assess the mediating effect of episodes of bullying in the past on the relationship between perpetration of bullying and externalizing symptoms. The significance of this mediating relationship was further verified with Sobel, Aroian, and Goodman statistics (32, 33). Thereafter, a two-step clustering algorithm was used to identify clusters of respondents based on their bullying behavior, strengths and difficulties. A log-likelihood model-based distance measure was used in the present analysis. This approach combines the clusters and evaluates the distance between them as the corresponding decrease in log-likelihood (34). First, all cases were sorted into pre-clusters, which were then clustered according to a hierarchical algorithm. Akaike’s information criterion (AIC) was then used to select the “best” cluster solution. The silhouette measure of cohesion and separation was used to assess the quality of the cluster, with a cut-off value of ≥0.5 considered as “good”.

Several approaches were used to minimize missing data. For example, respondents were given educational brochures and presentations to help them understand the importance of this study. They were asked to double-check their questionnaire to ensure no items were left unanswered. Finally, if there were any missing data, continuous values were replaced with the means of the scale for a given respondent, and values for categorical variables were replaced with the mode.

## Results

### Demographics

Out of 2,650 parents approached, a total of 2,325 provided consent for their child to participate in the survey. There was no rejection from the children and adolescents themselves, and the final overall response rate was 87.74%. Mean age of the respondents was 14.63 (2.87) years. Independent sample t-tests revealed that mean age in boys, i.e. 14.97 (3.58) years, was significantly older than in girls ($\bar{\text{x}}$ = 14.20, SD = 1.39). More than half of the respondents were males (n = 1301, 56.2%), and a majority reported that their mothers were housewives (n = 2,100, 90.7%). A high proportion of respondents reported that they had felt the need to ask for outside help in the preceding six months (n = 1,117, 48.2%), especially from their parents (n = 1,004, 43.4%).

### Prevalence of Bullying Behavior

A total of 615 (26.6%) respondents reported being bullied at school, and 415 (17.9%) away from school. The perpetration of bullying was reported by 430 (18.6%) participants at school and 376 (16.3%) away from school. Approximately one fourth of the participants (n = 559, 24.4%) reported being bullied by girls, and 516 (22.3%) by boys. A smaller number (n = 413, 17.8%) reported being bullied by a group more than once a week. Further exploration of the patterns of bullying revealed a total of 735 (31.70%) bully-victims. The experience of being bullied in the past showed a strong association with bully-victim behaviors (*X*_2_ = 1,298.19, P < 0.001, φ = 0.75). Student bullying at school showed a positive association with bullying away from school (ρ = 0.59, P < 0.001). Similar trends were noted for victimization behavior (ρ = 0.56, P < 0.001). Respondents who had been bullied in the past also scored significantly higher on the SDQ and its subscales than their counterparts who did not report any victimization experiences. Detailed demographic characteristics and patterns of bullying are presented in **Table 1**. The prevalence of history of victimization, perpetration and bully-victim experiences were significantly higher among children of mothers who were employed outside the home. Both boys and girls were more frequently bullied by other boys than by girls or groups (**Supplementary Table 1**).

**Table 1:** Demographic characteristics and trends in bullying among Pakistani schoolchildren.

Variables	Subcategories	Frequency (n)	Percentage (%)
Gender	Boy	1,301	56.2%
	Girl	1,014	43.8%
Mother’s profession	Housewife	2,100	90.7%
	Employed	215	9.3%
Have you felt the need to ask for outside help in the past six months?	No, I have not felt the need	600	25.9%
	I have considered getting outside help	599	25.9%
	I have sought outside help	1,117	48.2%
Preference for seeking help	Parents	1,004	43.4%
	Teachers	382	16.5%
	Relatives	73	3.2%
	Siblings	162	7.0%
	Friend	573	24.7%
	Other	122	5.3%
How often have you been bullied in school in the past six months?	Not at all	1,434	61.9%
	Less than once a week	267	11.5%
	More than once a week	259	11.2%
	Most days	356	15.4%
How often have you been bullied away from school (such as in tuition centers) in the past six months?	Not at all	1,651	71.3%
	Less than once a week	250	10.8%
	More than once a week	259	11.2%
	Most days	156	6.7%
How often have you bullied others in school in the past six months?	Not at all	1470	63.5%
	Less than once a week	416	18.0%
	More than once a week	212	9.2%
	Most days	218	9.4%
How often have you bullied others away from school (such as in tuitions centers) in the past six months?	Not at all	1,708	73.7%
	Less than once a week	232	10.0%
	More than once a week	157	6.8%
	Most days	219	9.5%
How often have girls bullied you?	Not at all	1,532	66.8%
	Less than once a week	200	8.7%
	More than once a week	161	7.0%
	Most days	398	17.4%
How often have boys bullied you?	Not at all	1,574	68.0%
	Less than once a week	226	9.8%
	More than once a week	276	11.9%
	Most days	240	10.4%
How often has a group bullied you?	Not at all	1,705	73.6%
	Less than once a week	198	8.5%
	More than once a week	163	7.0%
	Most days	250	10.8%
Have you told anyone about your experience so that they can help you?	Yes	1,646	71.1%
	No	670	28.9%
If yes please indicate who you told:	Parents	1,126	48.6%
	Teachers	389	16.8%
	Siblings	303	13.1%
	Friends	386	16.7%
	School	44	1.9%
	Other	68	2.9%

### Responses on the SDQ

According to these data, a total of 527 (22.8%) participants had borderline abnormal scores, and 479 (20.7%) had severe difficulties. According to SDQ subscale results, abnormal scores were seen in 600 (25.9%) participants for emotional problems, 583 (25.2%) for conduct problems, 195 (8.4%) for hyperactivity, 389 (16.8%) for peer problems, and 435 (18.8%) for poor prosocial skills. According to the results for the impact supplement scale, 299 (12.9%) participants had borderline scores and 902 participants (39.0%) had severe problems.

### Bullying, Distress and Help-Seeking Behavior

Most respondents (1,646, 71.1%) had told someone about their experience of being bullied, and a high proportion indicated that they had sought help from their parents (n = 1,126, 48.6%). **Supplementary Table 2** presents the association of bullying experiences with individual items on the impact supplement scale.

### Bullying and Emotional and Behavioral Difficulties

Linear regression analysis yielded a significant model predicting total scores on the SDQ scale (adj. R^2^ = 15%, F = 68.88, DF_total_ = 2,314, P < 0.001). According to this model, higher SDQ scores were associated with male gender, older age, and mother’s employment outside the home. Although a history of bullying correlated with higher SDQ scores, the strongest association was observed for children who reported both perpetration and victimization, followed by perpetration only and victimization only (**Table 2**). Similar trends were noted in the frequencies of distress reported by participants (adj. R^2^ = 8.5%, F = 36.64, DF_total_ = 2,314, P < 0.001).

**Table 2:** Patterns of bullying behaviors as predictors of SDQ scores.

Variables	B	Std. Error	Beta	t-value	P value
(Constant)	14.089	0.843		16.713	<0.001
Gender	−2.444	0.238	−0.212	−10.249	<0.001
Age	0.086	0.039	0.043	2.221	0.026
Mother’s profession	1.070	0.383	0.054	2.791	0.005
Bully victims	3.245	0.275	0.264	11.813	<0.001
Pure victims	1.582	0.343	0.095	4.604	<0.001
Pure perpetrators	2.121	0.379	0.113	5.590	<0.001

Being bullied in the past exerted significant mediation effects on the relationship between bullying others and externalizing symptoms (**Table 3**), as shown by hierarchal regression analysis (F = 108.96, df_total_ = 2,315, P < 0.001). These variables yielded significant effects in the Sobel test (test statistic = 6.32, SE = 0.07, P < 0.001), Aroian test (test statistic = 6.32, SE = 0.07, P < 0.001) and Goodman test (test statistic = 6.33, SE = 0.07, P < 0.001). In a similar manner (**Table 5**), being victimized in the past exerted significant mediation effects on the relationship between bullying others and internalizing symptoms (**Table 4**), as shown by hierarchal regression analysis (F = 108.96, df_total_ = 2,315, P < 0.001). These variables yielded significant effects in the Sobel test (test statistic = 6.74, SE = 0.06, P < 0.001), Aroian test (test statistic 6.73, SE = 0.06, P < 0.001) and Goodman test (test statistic = 6.74, SE = 0.06, P < 0.001).

**Table 3:** Controlling effects of bullying perpetration on the association between victimizing behavior and externalizing symptomatology.

Model		B	Std. Error	Beta	t-value	P value
1	(Constant)	4.975	0.146		34.060	<0.001
	Frequency of victimization	1.016	0.077	0.266	13.274	<0.001
2	(Constant)	4.605	0.157		29.413	<0.001
	Frequency of victimization	0.628	0.098	0.165	6.402	<0.001
	Frequency of perpetration	0.642	0.103	0.160	6.232	<0.001

**Table 4:** Controlling effects of bullying victimization on the association between victimizing behavior and externalizing symptomatology.

Model		B	Std. Error	Beta	t-value	P value
1	(Constant)	11.994	0.237		50.662	<0.001
	Frequency of perpetration	1.560	0.131	0.241	11.946	<0.001
2	(Constant)	11.247	0.253		44.386	<0.001
	Frequency of perpetration	0.750	0.167	0.116	4.501	<0.001
	Frequency of victimization	1.217	0.159	0.197	7.660	<0.001

### Cluster Analysis

A two-step clustering algorithm was used to group cases into two clusters. The overall quality of the clusters was rated as good, with a silhouette measure of cohesion and separation of 0.5. The first cluster—”normal behavior”—contained lower scores on all SDQ subscales. It represented 0% of bully-victims, 100% of the pure perpetrators and 100% of the pure victims. The second cluster—”psychopathological behavior”—contained significantly higher scores on all subscales except prosocial behavior. The SDQ subscale scores clustered 100% of bully-victims and excluded respondents with a history of victimization only or perpetration only (**Table 5** and **Supplementary Figure 1**). Thereafter, cluster analysis was done by introducing variables based on pure victims, pure perpetrators, or bully-victims, with similar results (**Supplementary Figure 2**).

**Table 5:** Mean (SD) scores on SDQ subscales in the two clusters.

SDQ subscales	Clusters			
		Low psychopathology n = 1581 (68.3%)	High psychopathology n = 735 (31.7%)	
**Children with history of bullying**	**Frequency**	**Percentage**	**Frequency**	**Percentage**
Bully victims	0	0%	735	100%
Perpetrators	239	15.1%	735	100%
Victims	315	19.9%	735	100%
Scores on SDQ scale	Mean	SD	Mean	SD
Emotional problems	4.08	1.93	4.58	1.94
Conduct problems	2.81	1.98	4.07	2.06
Hyperactivity	3.13	2.04	4.16	2.05
Peer problems	3.30	1.87	4.10	1.91
Prosocial behavior	6.67	1.80	5.60	2.15
Global score	13.32	5.56	16.92	5.31
Externalizing symptoms	5.94	3.41	8.23	3.33
Internalizing symptoms	7.38	2.99	8.68	2.92
Impact scores	10.04	3.63	12.31	4.00

## Discussion

Our survey results show a high prevalence of bullying victimization as well as perpetration among Pakistani schoolchildren. Almost all of the students who reported being bullied in the past also reported perpetrating bullying behaviors themselves. This behavior was associated with significant distress as shown by the responses on the SDQ impact supplement, and was also associated with severe behavioral problems as identified by the main SDQ.

An especially disturbing result is that almost 45.3% of the students reported being victims of bullying, while 42.1% were perpetrators of bullying and 31.2% were both victims and perpetrators. These figures are consistent with earlier surveys in Pakistan. For example, a high prevalence of verbal (57%) and physical abuse (33.7%) was reported among schoolchildren in the cities of Karachi, Lahore and Quetta (35). Similar prevalence rates of bullying behavior were reported by Zhu and Chan among Chinese schoolchildren (36). However, lower prevalence rates of bullying behaviors were reported among children in developed countries such as Australia, where the findings were attributed to better implementation of intervention programs that targeted bullying behaviors among schoolchildren (37).

Our data thus reveal a high prevalence of behavioral problems among Pakistani schoolchildren. Interestingly, there are no systematic reviews or metaanalyses summarizing the global prevalence of behavioral problems identified with the SDQ among schoolchildren. However, a systematic review by Salmanian et al. concluded that studies based on this instrument reported higher prevalence of conduct problems than other tools such as the Kiddie Schedule for Affective Disorders and Schizophrenia or clinical interviews used in the same populations (38). These authors also concluded that the SDQ might be useful as a screening tool, and that further psychiatric research should be done to ascertain the prevalence rate of behavioral problems among children (38). Therefore, the present findings should be interpreted with caution.

In the present study, more than 43% of the children scored in the abnormal range of the SDQ. Syed et al. reported similar prevalence rates of emotional and behavioral difficulties among children 7 to 11 years old in Karachi who completed the SDQ (39). These results are consistent with findings published by Goodman et al., who reported a high prevalence of emotional difficulties (10–15%) among children and adolescents in different settings such as rural Brazil and deprived British populations, and prevalence of 22% in urban Yemen and urban Brazil slum settings, 30–32% in Russia and Bangladesh, and 60% in rural Yemen (40). Our results are also consistent with those of Polanczyk et al. and Kovess-Masfety et al., who reported a significantly lower worldwide pooled prevalence of mental disorders compared to the present findings (41, 42).

The present findings show that children whose mother was employed outside the home had a higher prevalence of mental health problems and victimization by bullies at school than children from families in which the mother worked as a housewife. This highlights the patriarchal nature of Pakistani society, where working women may also be expected to shoulder a higher burden of child care than their male counterparts (43). This situation, coupled with a difficult work environment, lower wages, life stressors and the lack of day-care facilities for working women, acts as a double-edged sword that limits the ability of working mothers to provide care for their children.

Both being bullied and bullying others were associated with significant distress at school, during leisure and home activities, and in friendships, and higher SDQ scores. However, bullied victims reported more distress and more emotional and behavioral difficulties than bullies. It is usually believed that perpetrators of bullying exhibit externalizing symptoms (anger, criminality, and aggression), whereas victims of bullying exhibit internalizing symptoms (depression, anxiety, fear, and social withdrawal) (44–46). This belief is supported by Gini, who reported a higher propensity for psychosocial maladjustment and poor coping behavior among bullied schoolchildren than in their perpetrator counterparts (19). We believe that the higher prevalence of distress among victims of bullying may therefore be partly explained by two factors: a) victims experience comorbid externalizing and internalizing disorders, and b) a high proportion of these victims are bully-victims, who are themselves involved in perpetration behavior (37). This learned behavior is a consequence of the sociocognitive development of bullied children. After their experiences of being bullied by their peers, victims begin to believe in power symmetry and develop intolerance toward power differentials among peers; this in turn can lead to a higher prevalence of counterattacks (6). Thus, the victims themselves resort to the perpetration of bullying, and contribute to the resulting vicious circle of perpetration and victimization, adding further to their psychological injuries (6).

In the present study, a two-step cluster algorithm was used to examine clusters and dimensions of psychopathologies (assessed with the SDQ) among respondents. These results indicated that perpetrators, victims and bully-victims regressed to the same clusters when psychopathologies were taken into account. This finding confirms the homogenous nature of psychopathologies among adolescents in Pakistan, once the large overlap between victimization and perpetration behavior is controlled for. As such, it is a relatively novel finding, given that previous studies with cluster analysis classified bullying subtypes rather than behavioral and emotional problems. For example, one study of bullying behavior subtypes identified unwanted sexual and internet solicitation subgroups, and another identified uninvolved adolescents, victims, verbal bullies, bully-victims, and physically aggressive bullies, based on social support and skills status and social behavior (25). A similar conclusion can be drawn from the mediation analyses, which challenge the prevalent notions that pure victimization is categorically associated with internalizing disorders and pure perpetration with externalizing disorders. We feel that future studies should conceptualize the association between pediatric behavioral disorders and bullying behaviors from a dimensional approach (47, 48).

The present results and analysis have several practical implications. They underscore the synergistic effect of perpetration and victimization behavior among young people, leading to a plethora of emotional and behavioral psychopathologies that persist later in life. Moreover, these strengths and difficulties tend to cluster together. Therefore, intervention programs should be multimodal and comprehensive, targeting both externalizing disorders such as aggression and internalizing disorders such as depression among youth. These intervention programs should also implement psycho-educational knowledge of victimization, perpetration and bully-victim behavior together (24, 25). Our results are also corroborated by a study that found a similar clustering of physical health behaviors among different forms of bullying (27). According to that research, Dutch adolescents who showed bullying behavior had high rates of risk-prone behavior, screen use, sedentary lifestyle, higher body mass index values, problematic self-efficacy and problematic SDQ scores (27).

### Strengths of the Study

The present study has several strengths. Our large sample of Pakistani schoolchildren included respondents in seven cities across two provinces of Pakistan, which provided high statistical power for the data analyses. Despite the use of a convenience sampling method, the approach used to seek voluntary participation together with the high response rate make our findings potentially generalizable to the Pakistani population of schoolchildren in the same age group. The participating schools in this study were situated in small cities catering to their respective populations. But a large proportion of these children had a rural background, which increases the generalizability of our findings to the rural population as well.

### Limitations

The cross-sectional nature of this study limits inferences related to temporality and causality. These hypotheses should therefore be explored in future studies of longitudinal cohorts. In addition, the use of a self-administered instrument may have led to some recall bias; moreover, no collateral information was obtained from the children’s parents or teachers.

In conclusion, the prevalence of bullying perpetration and victimization behaviors among Pakistani schoolchildren was very high and was associated with behavioral difficulties. This study also provides insights into the association between childhood psychopathologies, different patterns of bullying behavior, and the mediators governing them. Bullying perpetration was associated with externalizing symptoms, whereas being a victim was associated with both externalizing and internalizing psychopathologies. The higher prevalence of distress among victims of bullying can be explained by comorbid externalizing and internalizing disorders, and by a greater tendency of victims to engage in bullying perpetration themselves as a learned behavior.

Our findings have several implications for Pakistani policy makers and researchers. We emphasize the need to understand the patterns of behavioral difficulties in order to design effective anti-bullying initiatives, psychosocial counseling procedures, and school-based mental health services.

## Ethics Statement

Ethical approval for this study was granted by the Peoples University of Medical and Health Sciences for Women, Nawabshah, and it was found to meet the ethical criteria of the Declaration of Helsinki (1964) and comparable standards. The informed consent form and a detailed brochure outlining the objectives of the study were mailed to each participant’s parents. They were assured that all data would be anonymous and that no individual findings would be reported.

## Author Contributions

SN and AW conceived and designed the study, supervised data collection, analyzed the data and interpreted it, and wrote the initial draft of the manuscript. ZS, WA, MW, JR, and TA collected the data and edited the draft extensively. All authors reviewed the final draft and approved it for publication.

## Conflict of Interest

The authors declare that the research was conducted in the absence of any commercial or financial relationships that could be construed as a potential conflict of interest.
