# Supplementary material for: Trends in Bullying and Emotional and Behavioral Difficulties Among Pakistani Schoolchildren: A Cross-Sectional Survey of Seven Cities
Source: Front Psychiatry. 2020 Jan 17;10:976. doi: 10.3389/fpsyt.2019.00976 (PMC6978956; doi:10.3389/fpsyt.2019.00976)

Supplementary figure 1: Graphical representation of clusters identified in two-step cluster analysis


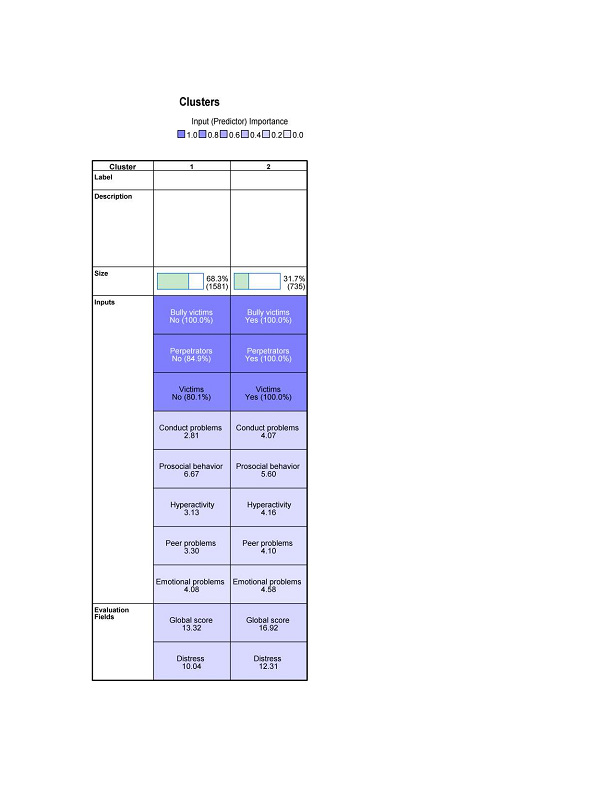


Supplementary figure 2: Graphical representation of clusters based on pure perpetrators, pure victims, and bully-victims


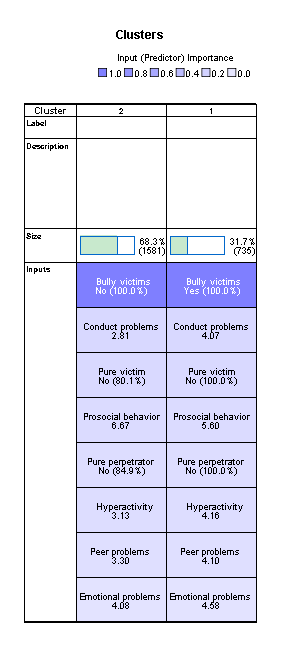

Supplement: Supplementary file 1 [file DataSheet_1.docx]
