# Supplementary material for: Trends in Bullying and Emotional and Behavioral Difficulties Among Pakistani Schoolchildren: A Cross-Sectional Survey of Seven Cities
Source: Front Psychiatry. 2020 Jan 17;10:976. doi: 10.3389/fpsyt.2019.00976 (PMC6978956; doi:10.3389/fpsyt.2019.00976)
Supplement: Supplementary file 2 [file DataSheet_2.docx]

Supplementary Table 1: Association between history of bullying and mother’s employment status

|  | | Housewife | | Employed | | Chi-squared | P |
| --- | --- | --- | --- | --- | --- | --- | --- |
|  | | Frequency | Percentage | Frequency | Percentage |  |  |
| Victims | No | 1188 | 56.6% | 77 | 35.8% | 33.91 | <0.001 |
|  | Yes | 912 | 43.4% | 138 | 64.2% |  |  |
| Perpetrators | No | 1261 | 60.0% | 80 | 37.2% | 41.74 | < 0.001 |
|  | Yes | 839 | 40.0% | 135 | 62.8% |  |  |
| Bully victims | No | 1483 | 70.6% | 97 | 45.1% | 58.54 | <0.001 |
|  | Yes | 617 | 29.4% | 118 | 54.9% |  |  |

Supplementary Table 2: Gender distribution of bullying experiences

| Variables | | Boys | | Girls | |
| --- | --- | --- | --- | --- | --- |
|  | | Frequency | Percentage | Frequency | Percentage |
| Do girls bully you? | No | 747 | 57.4% | 785 | 77.3% |
|  | Yes | 554 | 42.6% | 230 | 22.7% |
| Do boys bullies you? | No | 619 | 47.6% | 955 | 94.1% |
|  | Yes | 682 | 52.4% | 60 | 5.9% |
| Do groups bully you? | No | 784 | 60.3% | 921 | 90.7% |
|  | Yes | 517 | 39.7% | 94 | 9.3% |

Supplementary Table 3: Impact scores as assessed by the Strengths and Difficulties Questionnaire

|  | | History of bullying experiences | | | |
| --- | --- | --- | --- | --- | --- |
|  |  | No | | Yes | |
|  |  | Count | Column N % | Count | Column N % |
| Overall, do you think that you have difficulties in one or more of the following areas: emotions, concentration, behavior or being able to get on with other people? | No | 384 | 37.4% | 347 | 26.9% |
|  | Yes, minor difficulties | 495 | 48.2% | 625 | 48.5% |
|  | Yes, definite difficulties | 92 | 9.0% | 232 | 18.0% |
|  | Yes, severe difficulties | 56 | 5.5% | 85 | 6.6% |
| How long have these difficulties been present? | Less than a month | 766 | 74.6% | 744 | 57.7% |
|  | 1-5 months | 130 | 12.7% | 287 | 22.3% |
|  | 6-12 months | 38 | 3.7% | 105 | 8.1% |
|  | Over a year | 93 | 9.1% | 153 | 11.9% |
| Do the difficulties upset or distress you? | Not at all | 378 | 36.8% | 314 | 24.4% |
|  | Only a little | 452 | 44.0% | 604 | 46.9% |
|  | Quite a lot | 127 | 12.4% | 270 | 20.9% |
|  | A great deal | 70 | 6.8% | 101 | 7.8% |
| Home life | Not at all | 612 | 59.6% | 535 | 41.5% |
|  | Only a little | 242 | 23.6% | 429 | 33.3% |
|  | Quite a lot | 117 | 11.4% | 209 | 16.2% |
|  | A great deal | 56 | 5.5% | 116 | 9.0% |
| Friendships | .00 | 0 | 0.0% | 1 | 0.1% |
|  | Not at all | 627 | 61.1% | 515 | 40.0% |
|  | Only a little | 285 | 27.8% | 468 | 36.4% |
|  | Quite a lot | 74 | 7.2% | 222 | 17.2% |
|  | A great deal | 41 | 4.0% | 81 | 6.3% |
| Classroom learning | Not at all | 637 | 62.0% | 525 | 40.7% |
|  | Only a little | 187 | 18.2% | 375 | 29.1% |
|  | Quite a lot | 121 | 11.8% | 247 | 19.2% |
|  | A great deal | 82 | 8.0% | 142 | 11.0% |
| Leisure activities | Not at all | 623 | 60.7% | 579 | 44.9% |
|  | Only a little | 201 | 19.6% | 326 | 25.3% |
|  | Quite a lot | 117 | 11.4% | 241 | 18.7% |
|  | A great deal | 86 | 8.4% | 143 | 11.1% |
| Do the difficulties make it harder for those around you (family, friends, teachers, etc.)? | Not at all | 762 | 74.2% | 722 | 56.0% |
|  | Only a little | 181 | 17.6% | 353 | 27.4% |
|  | Quite a lot | 54 | 5.3% | 156 | 12.1% |
|  | A great deal | 30 | 2.9% | 58 | 4.5% |
